# Cortical beta oscillations help synchronise muscles during static posture holding in healthy motor control✰

**DOI:** 10.1016/j.neuroimage.2024.120774

**Published:** 2024-08-03

**Authors:** Thomas G. Simpson, William Godfrey, Flavie Torrecillos, Shenghong He, Damian M. Herz, Ashwini Oswal, Muthuraman Muthuraman, Alek Pogosyan, Huiling Tan

**Affiliations:** a Medical Research Council Brain Network Dynamics Unit, Nuffield Department of Clinical Neurosciences, University of Oxford, Oxford, UK; b Movement Disorders and Neurostimulation, Department of Neurology, Focus Program Translational Neuroscience (FTN), University Medical Center of the Johannes Gutenberg-University Mainz, Mainz, Germany; c Neural Engineering with Signal Analytics and Artificial Intelligence (NESA-AI), Department of Neurology, Universitätsklinikum Würzburg, Würzburg, Germany

**Keywords:** Beta oscillation, Posture holding, High-density EEG EMG, Corticomuscular coherence, Intermuscular coherence

## Abstract

How cortical oscillations are involved in the coordination of functionally coupled muscles and how this is modulated by different movement contexts (static vs dynamic) remains unclear. Here, this is investigated by recording high-density electroencephalography (EEG) and electromyography (EMG) from different forearm muscles while healthy participants (*n* = 20) performed movement tasks (static and dynamic posture holding, and reaching) with their dominant hand. When dynamic perturbation was applied, beta band (15–35 Hz) activities in the motor cortex contralateral to the performing hand reduced during the holding phase, comparative to when there was no perturbation. During static posture holding, transient periods of increased cortical beta oscillations (beta bursts) were associated with greater corticomuscular coherence and increased phase synchrony between muscles (intermuscular coherence) in the beta frequency band compared to the no-burst period. This effect was not present when resisting dynamic perturbation. The results suggest that cortical beta bursts assist synchronisation of different muscles during static posture holding in healthy motor control, contributing to the maintenance and stabilisation of functional muscle groups. Theoretically, increased cortical beta oscillations could lead to exaggerated synchronisation in different muscles making the initialisation of movements more difficult, as observed in Parkinson’s disease.

## 1 Introduction

Coordination of functionally coupled muscles is a key aspect of healthy motor control. Co-activation of different muscles is required for static posture holding, whereas individual muscle control may be required for the initialisation of movement. This is associated with changes in beta oscillations in the sensorimotor cortical-basal ganglia network, which typically reduces during movement initiation and execution, and rebounds to a higher than previous level following the finish of a movement (He et al., 2020; Jurkiewicz et al., 2006; Pfurtscheller et al., 2003). Notably, elevations in beta power have been shown to cause slowing of spontaneous movement, as well as an increase in the number of corrective responses to postural perturbation (Gilbertson et al., 2005). The role of this activity is important in movement disorders, where increased beta oscillations in the basal ganglia are seen as a putative biomarker for bradykinesia and rigidity in Parkinson’s disease (Jenkinson and Brown, 2011; Little et al., 2012; Pogosyan et al., 2010). These observations have contributed to the hypothesis that synchronisation in beta band oscillations within the sensorimotor network is expressed more strongly if the maintenance of the status quo is intended or predicted (Engel and Fries, 2010). However, it is still not clear how sensorimotor cortical oscillations are involved in the coordination of functionally coupled muscles and how the effect of cortical oscillations on the muscle activities changes with different movement requirements and contexts (static vs dynamic contractions).

Intermuscular coherence (IMC) has been used to investigate functional coordination and synergy between different muscles, where corticomuscular coherence (CMC) reflects the interaction between the cerebral cortex and the muscle activities. Previous research demonstrates that during steady state isometric contraction, beta band CMC is increased between the sensorimotor cortex and contracting muscle (Gwin and Ferris, 2012; Jacobs et al., 2015; Kristeva et al., 2007). This has also been postulated to be reflective of synergistic control strategies, such that beta CMC increases during muscle co-contraction, but is reduced when individual control of different muscle groups is required (Reyes et al., 2017). On the other hand, increases in the CMC in the gamma band (>30 Hz) have been reported during strong contraction (Brown et al., 1998) and during dynamic force output (Omlor et al., 2007).

Recent studies have challenged the sustained nature of rhythmic beta activity, demonstrating a transient component to beta that occurs in short, burst like, events known as beta bursts (Bartolo and Merchant, 2015; Feingold et al., 2015; Sherman et al., 2016; Shin et al., 2017; Tinkhauser et al., 2017a, 2017b). However, there is limited research on the effect of those transient events of increased cortical beta oscillations on muscle activity and muscle coordination. In a recent study (Echeverria-Altuna et al., 2022) it was demonstrated that cortical beta bursts were associated with corresponding increases in the muscle beta activity during an isometric force generation task. How cortical beta bursts modulate intermuscular coherence, and whether this effect is different for tasks that have different muscle coordination requirements remains unknown.

This study aims to address these questions by measuring high-density EEG and EMG from multiple muscles, in healthy participants while performing a motor task involving static posture holding, active perturbation resisting, and cued reaching movements on the Kinarm End-Point robotic platform (BKIN Technologies Ltd, Canada). Beamforming source reconstruction was used to extract the signal from the precentral gyrus on the sensorimotor cortex to improve the signal-to-noise ratio of the original EEG data. This investigation aims to address the role of sensorimotor beta oscillations in coordinating functionally coupled muscles, and how this varies during different types of motor task. These roles could be significant in understanding the pathology of movement disorders such as Parkinson’s disease.

## 2 Methods

### 2.1 Ethical approval

This experiment was approved by the University of Oxford Research Ethics Committee.

### 2.2 Participants

A total of 20 participants (10 males and 10 females) aged between 19 and 62 (*μ* = *22.35yrs, SD* = *9.36yrs*) were recruited via college wide invitation. 18 participants were right-handed and 2 were left-handed based on the Edinburgh Handedness Questionnaire. All participants had normal or normal-corrected vision. They gave informed consent prior to the study and received monetary compensation for their time. Participants were able to understand and complete the task without issue.

### 2.3 Experimental setup

The task was developed and implemented on the Kinarm robotic system using MATLAB, Simulink, and proprietary Kinarm software.

**Fig. 1:**
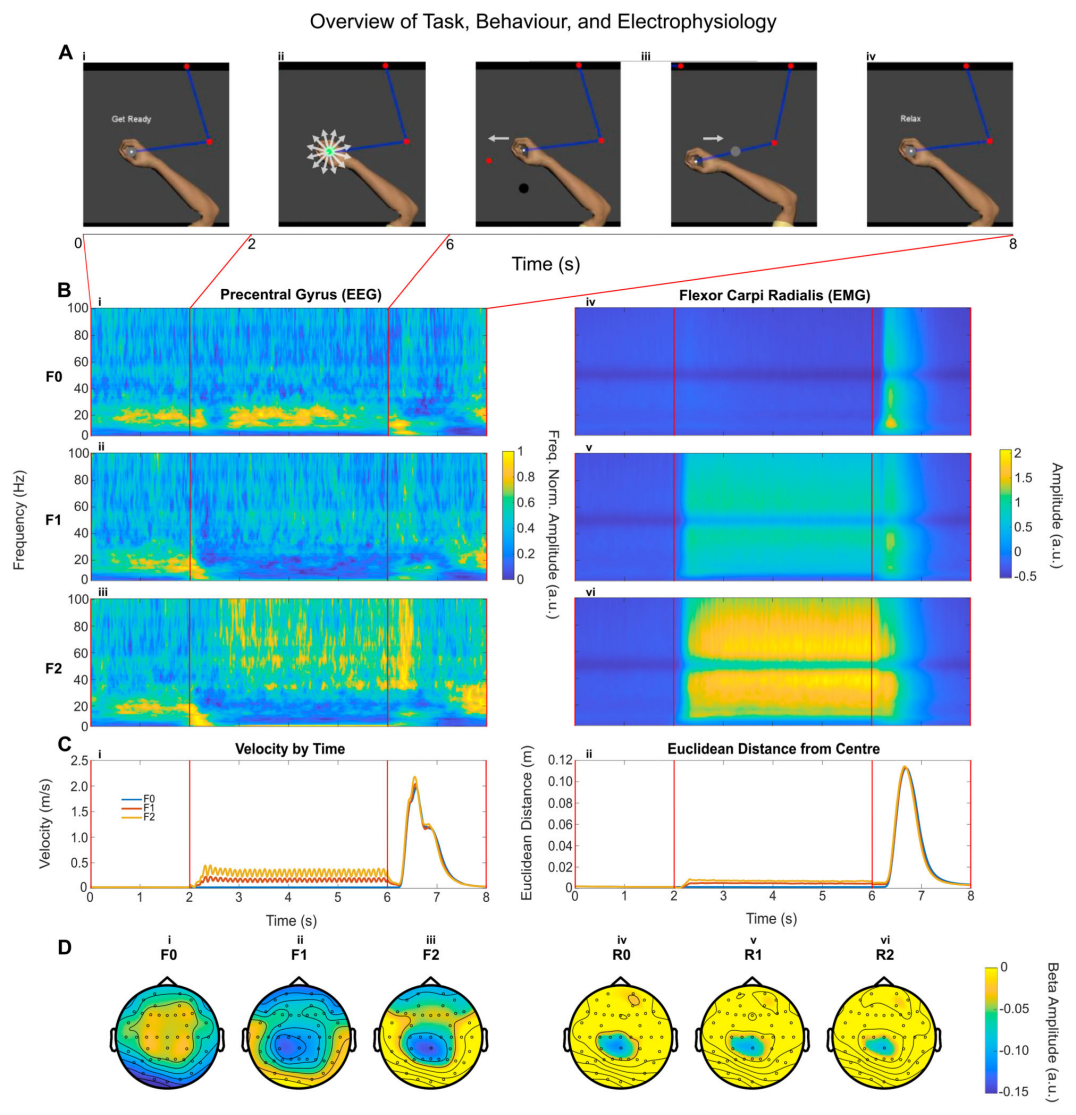
Study overview with A (i-v) demonstrating different stages of the task. The arm is added here for visualisation, but the cursor is presented to the user throughout the task. Each trial consists of 5 phases: (i) ‘Get ready’; (ii) ‘Steady’ or ‘hold’, when the manipulandum produces perturbation forces in different directions; (iii) ‘Go’, when the red target cue prompts participants to move the cursor to the target as fast as possible, followed by a return to the centre (iv) ‘Relax’, when waiting for the next trial. The accompanying electrophysiological activity is demonstrated (B) for the EEG (precentral gyrus) and EMG (the flexor carpi radialis) averaged across all participants for all force conditions (F0, F1, F2). The accompanying behavioural metrics of velocity and Euclidean distance of the cursor from the centre position are also depicted in C. Finally, the average topographical plots at beta (15–35 Hz) for the different force conditions F0, F1, and F2, are given in D with i, ii, iii, during steady holding phase and iv, v, vi during reaching phase.

During the task, participants were sat at the Kinarm, facing a screen on which the position of the manipulandum was displayed as a cross. Each trial of the task had four phases: Ready, Steady, Go, and Relax before the next trial start. When a ‘Get Ready’ sign appeared on the screen (Fig 1A), the participants were instructed to move the manipulandum to the centre position so that the cursor (diameter = 1 cm) was inside a circle (diameter = 4 cm) displayed in the centre of the screen. After the cursor stayed within the required place for two seconds (fixed), the trial entered the second phase (Steady phase) which lasted for between 4 and 5 s (randomised), during which there would be either no perturbation or perturbations of different force levels (randomised across trials). The participants were asked to keep the manipulandum steady in place so that the cursor was within the target circle in this phase no matter whether there was force perturbation or what level the force perturbation was (Fig 1B). The perturbation force was implemented with the Kinarm by pulling the manipulandum from its centre position to a random location in a circle around the centre point (5 cm away) with a specified force. The pulling force was set constant within each trial, applied every 100 ms and the direction of the pulling force was randomised, although it could not return to a quadrant more than twice on successive occasions. There were three force levels for different trials: F0, where no force was applied and the participant was holding the handle in the centre position (static posture holding); F1, where 5 % of the recorded maximal voluntary gripping force (MVC) was applied; and F2, where 10 % of the recorded MVC was applied.

Following the perturbation phase, the forces were removed, and a reach cue was presented as another target circle either on the left or right of the screen, upon which the user was expected to move the manipulandum as fast as possible (in terms of both response time and movement velocity), so that the cursor reached the target (Fig. 1A iii). Once the target was reached, they were required to return the cursor back to the centre circle (Fig. 1A iii), ending the trial. During the inter-trial interval (4−5 s), the participant could relax and was free to move the manipulandum around (Fig. 1 iv).

In total there were 36 trials per block and a total of 10 blocks with 3 force conditions, making a total of 120 trials per force condition (360 trials total) per participant including 2 reaching directions, left and right.

The MVC was measured for each participant using a dynamometer. Each individual was instructed to grip with maximum sustainable intensity for a duration of 10 s, which was averaged and recorded. This process was repeated 5 times and averaged to ensure that participants could maintain the applied force for the duration of the experiment.

### 2.4 Recordings

Cortical neural activity was measured using a 64-channel wet electrode EEG cap and a TMSi-SAGA amplifier (both provided by TMSi, Netherlands), at a sampling frequency of 2048 Hz. EMGs were simultaneously recorded using the same amplifier with the electrodes placed on four separate locations on the forearm in bipolar configuration (rather than being constructed post-hoc) for each muscle: *flexor carpi radialis (Flx1), flexor digitorum superficialis (Flx2), extensor carpi ulnaris (Ext1)*, and *extensor digitorum (Ext2)*. The ground electrode was placed on the participants wrist.

Several metrics related to the movements of the manipulandum, including the position and velocity, as well as the force being applied, were recorded within the Kinarm environment.

The Kinarm and TMSi-SAGA recording systems were synchronised using a Kinarm generated voltage pulse output that used an event dependent amplitude to describe different events occurring in the task. For instance, the beginning of the task, when the perturbations began, when the reach cue was displayed, when the manipulandum reached the cue, amongst other events. This output was fed back into the Kinarm, as well as into the TMSi-SAGA recording system, so that both systems recorded the output signal, hence allowing for synchronisation.

### 2.5 Data processing

#### 2.5.1 Behavioural measurements

The position and velocity of the manipulandum was registered by the Kinarm software at a frequency of 1000 Hz.

The reaction time and the maximal displacement for the reaching movements was quantified based on the manipulandum position. Reaction time is defined as the time when the displacement of the manipulandum reached 10 % of the distance to the target.

#### 2.5.2 EEG and EMG analysis

To reduce noise and extract oscillations specific to the sensorimotor cortex, a spatial filtering technique known as linear constrained minimum variance (LCMV) (Muthuraman et al., 2020; Veen et al., 1997) and often referred to as beamforming (Veen and Buckley, 1988) was utilised. The spatial filter attenuates the signals from other locations and allows signals generated from a particular location in the brain, for a certain frequency band. The detailed description of the forward and inverse solutions is provided elsewhere (Muthuraman et al., 2008, 2010). Structural magnetic resonance images (MRI) are used for extracting the surfaces of the layers such as the scalp, skull, and brain, as opposed to individual scans. The conductivity value was 0.8 S/m for the scalp and brain, and 0.008 S/m for the skull (Muthuraman et al., 2015). Based on this forward model, various locations in the brain are specified in 3D coordinates allowing for selection of specific brain areas as the target source, by employing the spatial filter to extract the source time series. The cortical mesh surfaces here were created using FreeSurfer Version 4.0.1 (Dale et al., 1999; Fischl et al., 1999, 2001) from an average of 27 T1 scans of the same subject (Tzourio-Mazoyer et al., 2002). The surfaces were inflated to a sphere and downsampled using an octahedron (8196 vertices) or an icosahedron (5124 and 20,464 vertices) equally subdivided to achieve highly tessellated surfaces. The focus of this study is the precentral gyrus contralateral to the hand used to perform the task.

All signal processes were conducted in MATLAB (version 2019b). The data was pre-processed with a 100 Hz low pass filter, a 1 Hz high pass filter (both second order, two-pass Butterworth filters), and a 50 Hz notch filter to eliminate line noise. Continuous wavelet transform (CWT) was used for time-frequency decomposition with a Morlet wavelet of 10 cycles and a standard deviation of 3. The amplitude of each frequency band at different time points was calculated by taking the absolute value of the complex output.

The average amplitude for different frequency bands, at different time windows of the task, and for different task conditions were then qualified and compared. Beta band activity was defined within the frequency range of 15–35 Hz, and Gamma band was defined as 55–90 Hz. Beta bursts were subsequently defined as time periods where the beta amplitude exceeded its 75th percentile for a minimum of 100 ms. This 75th percentile is calculated based on the whole block (pooling all data across all force conditions together), which includes 12 trials of each force condition (of which there are 3, for a total of 36 trials per block). This means that the 3 force conditions have the same raw threshold for bursts. For comparison, ‘no burst’ periods were also extracted, which were defined as epochs when the cortical beta amplitude remained below the 50th percentile for a duration of at least 500 ms with the absence of a preceding beta burst (75th percentile) for a period of at least 500 ms. The amplitude of each individual frequency (per 1 Hz) was z-scored over each block for each participant using the standard formula:

$$\text{Z}=\frac{x-\mu }{\sigma }$$

and then mean averaged over the time period and frequency band in question.

#### 2.5.3 Corticomuscular coherence (CMC) and intermuscular coherence (IMC)

The phase−locking value (Aydore et al., 2013; Celka, 2007) was used to calculate corticomuscular coherence and intermuscular coherence. This was to compute the phase consistency between the motor cortex and the forearm muscles, as well as between the different muscles in the forearm. The PLV provides estimates of synchrony independent of the amplitude of oscillations. This is in contrast to measures of coherence where phase and amplitude are intertwined (Uhlhaas et al., 2010). In addition, phase synchrony is used in this study because some literature suggests it is better used for short duration events such as beta bursts (Bowyer, 2016). To calculate PLVs, the signals of interest were first band-pass filtered using a digital IIR filter, prior to Hilbert transformation. The instantaneous phase of each signal at each time point was extracted, and the phase difference between the signals were calculated. The vector strength of the phase difference was computed using a sliding window technique with a fixed window length of 250 ms period, with 125 ms before the sample and 125 ms after. The value at each time point is the vector strength of the phase difference over this 250 ms window. This was computed over the entire block and then averaged over accepted trials or intervals of interest (which varied depending on the participant). This procedure was repeated for each frequency band to generate a time-frequency coherence plot for each beta burst. The mean was found for each participant, before finally computing the mean across all participants (Fig. 3).

#### 2.5.4 Trial rejection

When the effect of the beta burst was investigated, the number of ‘trials’ is the number of beta bursts. The number of beta bursts detected in each condition is reported in Section 3.3. First, trials with the timeseries extracted precentral gyrus signal amplitude exceeding 6 standard deviations above the block mean were rejected. Secondly, the data was band-pass filtered between 15–35 Hz to extract the beta activity, and any remaining trial with value exceeding 4 standard deviations above the block mean amplitude of the filtered data was removed.

A further trial rejection step was included for the behavioural and EEG analyses. This was done by a visual and statistical inspection to confirm that the task was completed to a satisfactory standard, and that the EEG data had no obvious artefacts (such as movement, clear ocular artefact, or jaw clenching/muscular artefact). This resulted in 37.2 ± 12.8 trials (of 360) further rejected for each participant in the final analyses.

### 2.6 Statistics

There were two main statistical tests utilised in the study. The first was the repeated measures ANOVA, which was adopted when there were more than two groups. The second was the Wilcoxon Signed Rank test for post-hoc testing, and for when there were two groups. As it is a non-parametric test it is more robust to the small sample sizes in this work. Effect size is reported as eta squared (η^2^) in the ANOVA and rank-biserial correlation (*r*) in the Wilcoxon Signed Rank test. Furthermore, corrections for multiple comparisons is implemented with the Holm-Bonferroni procedure.

To test whether the EMG amplitude, CMC, and IMC were significantly different during cortical beta burst vs no-burst, permutation-based cluster analysis was employed. For IMC, this was implemented by generating a paired *t*-test plot between the two conditions of burst and no-burst, where values exceeding the threshold t-statistic 1.645 were included in the cluster, which was selected as it includes the top 5% of values. For CMC the same process is utilised, except that instead of using ‘no-burst’ observations, the cortical signal was kept the same and the

EMG data was randomly shuffled to generate a comparison. For the data, t-statistics for the clusters were determined, as well as the sizes of the clusters in pixels, with the largest values selected as the largest cluster. Then, the null distribution was generated by randomly swapping the pairings in the paired samples *t*-test, where the largest t-statistic and cluster size were recorded for each permutation, which created a set of possible cluster sizes under the null hypothesis. Finally, two final p-values were generated, one for the cluster size and one for the accumulated t-statistic, computed by comparison with the null distribution. Only the overall p-value from the accumulated t-statistic method is reported here, because there were no results that changed between the two methods.

### 2.7 Reaching movements

Reaching movements were added to the paradigm to compare the average beta power and the effect of cortical beta bursts on muscle activities during static posture holding, perturbation resisting and voluntary movements. However, due to the limited number of trials, short duration of ‘reaching movement’ in each trial, and the probability of beta bursts reduced during reaching movements, only very small number of beta bursts are observed during reaching movements per participant. Therefore, concrete conclusions about the effect of beta bursts on the muscles during reaching movements were not possible. Thus, the analysis is focused on the effect of bursts during the ‘holding’ phase only.

## 3 Results

This study aimed to investigate the role of cortical beta oscillations on motor control in healthy participants. To achieve this, an experiment was developed on the Kinarm involving a task with 3 force conditions as described in detail in Methods 2.3. Behavioural metrics from the different force conditions are given in Supplementary Fig 1. The results are organised into 4 main sections: firstly, the effect of the perturbation forces on cortical and muscle activities is analysed. The next two sections aim to evaluate the effect of cortical beta bursts on the IMC and CMC, further arguing that the observed effect is dependent on the nature of the movements. Finally, the effect of beta burst is demonstrated as frequency specific, as gamma bursts do not demonstrate such an effect, despite an established link with motor control.

### 3.1 Perturbation force conditions modulated cortical activities and their connectivity with muscle activities during the ‘Steady’ phase

The participants modulated muscle activities during the ‘Steady’ phase according to the perturbation force levels, as shown in the time-frequency plot of the flexor carpi radialis (Flx1) (Fig. 1B iv, v, and vi). Overall muscle activities increased when the perturbation forces increased. Despite the effort of the participant to maintain the position of the manipulandum in the centre during the steady phase, the velocity of the manipulandum (Fig. 1C i), and its Euclidean distance from the centre (Fig. 1C ii), were modulated by force level (*velocity: F(1.02, 19*) = *542.58, p <.001*, η*2* = *0.97, Euclidean distance: F(1.46, 19*) = *535.28, p <.001*, η*2* = *0.98*), because the greater perturbation force was causing the participant to compromise stability (*velocity and Euclidean Distance: F0 v F1, F0 v F2, F1 v F2: Z*= −*3.92, p < 0.001, r* = *0.88*).

These tests return the same results as all observations were higher in one condition when compared to the other. The ‘oscillatory pattern’ in the manipulandum velocity is because the perturbation was applied every 100 ms.

Cortical activities in the contralateral precentral gyrus were modulated by the task phase and perturbations force levels, as shown in the average time-frequency plot (Fig. 1B i, ii, and iii). In particular, beta band activities (15 − 35 Hz) remained high during the ‘Steady’ phase in the F0 condition (which is static posture holding without any perturbation) after a brief desynchronisation following the trial start visual cue. However, there was sustained beta desynchronisation during the ‘Steady’ phase in F1 and F2 condition, when the participants need to actively resist perturbations. Beta was also reduced during reaching movements. The beta desynchronisation seemed to focus on the contralateral sensorimotor areas during both ‘Steady’ and ‘Reach’ phase as shown in the topographical plots (Fig. 1D i, ii, iii, for ‘Steady’, and iv, v, vi for ‘Reach’ phases).

**Fig. 2:**
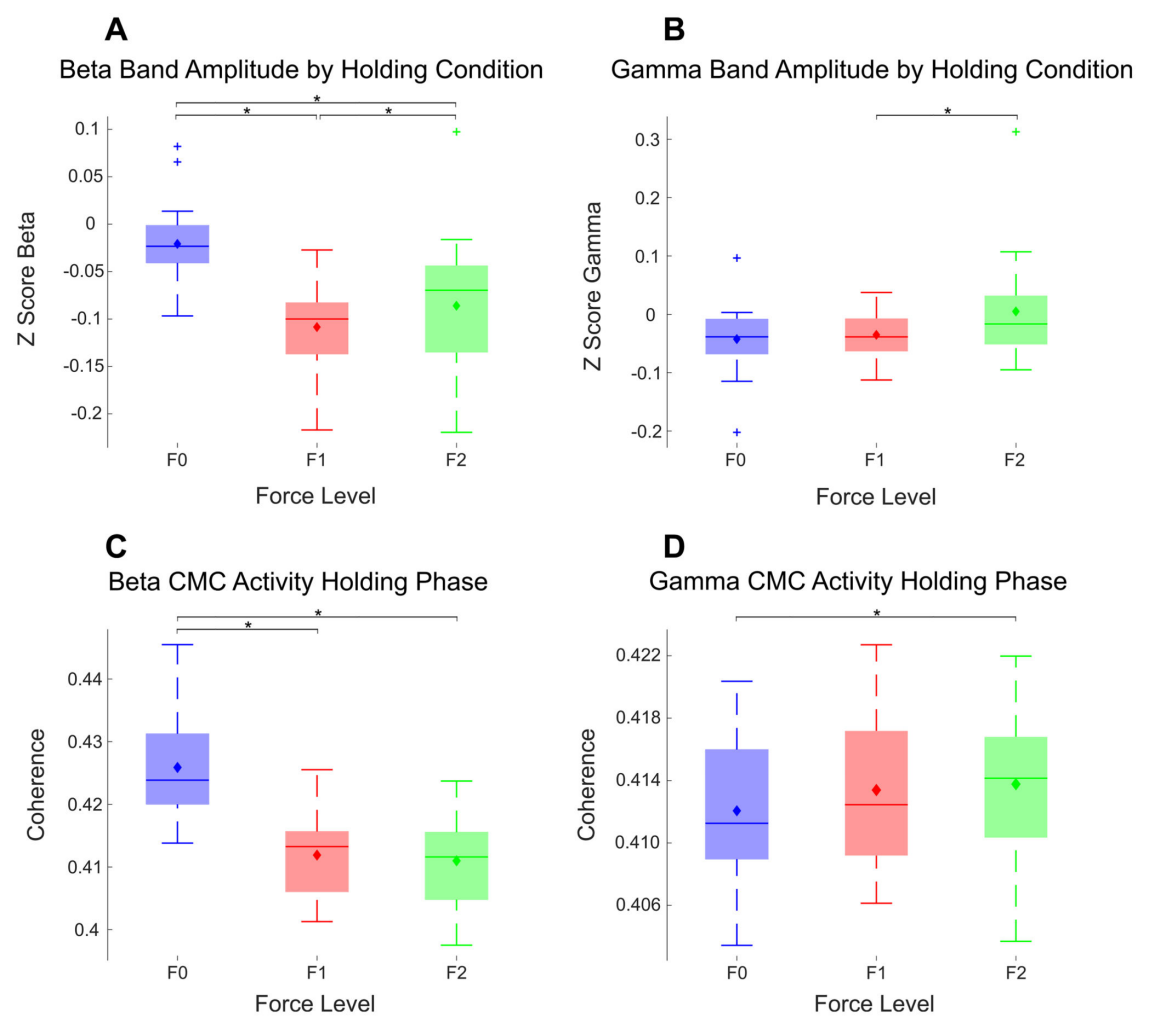
Cortical oscillations in the beta (15−35 Hz, A) and gamma (55−90 Hz, B) band, as well as the cortical-muscular coherence in the beta (C) and gamma (D) band during the steady holding phase were modulated by the perturbation force. Oscillations from the precentral gyrus contralateral to the performing hand were considered here. For CMC (quantified as PLV), the CMC between the contralateral precentral gyrus and all recorded EMGs were first averaged for each participant before the statistical test. The bars and error bars show the median average and standard error respectively across all participants, while the diamond demonstrates the mean. * indicates a significant difference in the post-hoc Wilcoxon signed rank test.

The average amplitude for different frequency bands was quantified (alpha: 8Hz-12 Hz; beta: 15Hz-35 Hz; gamma: 55Hz-90 Hz) during the ‘Steady’ phase (full length of the steady part of the trial was used). ANOVA analysis confirmed significant effect of the perturbation force on the average amplitude of beta oscillations (*F(1.14, 19*) = *12.93, p* = *0.001*, η*2* = *0.44*). As shown in Fig. 2A, beta activities were higher when there was no force perturbation (F0 condition), compared to the condition with unpredictable force perturbation, which required active movements to resist the perturbations (*F0 v F1: Z* = *3.58, p <.001, r* = *0.80, F0 v F2: Z* = *2.92, p =.008, r* = *0.65*), although, high perturbation elevated cortical beta amplitude above low perturbation *(F1 v F2: Z* = −*2.50, p=.01, r* = −*0.56*). Gamma oscillation was not significantly modulated by the perturbation forces (*F(1.11, 19) = 2.66, p* = *0.12*, η*2* = *0.13*) although there may be a small effect with the largest gamma amplitude occurring during the condition with largest force perturbations (Fig. 2B).

Average CMC and IMC in the beta and gamma frequency during the stable ‘Steady’ phase were also evaluated during the steady phase and compared between different force perturbation conditions. Beta band IMC averaged across all muscle pairs did not significantly change with perturbations forces (*F(1.04, 19*) = *2.37, p* = *0.14*, η*2* = *0.11*), while beta band CMC reduced with perturbations forces (Fig. 2C) (*F(1.24, 19) = 49.52, p < 0.001*, η^2^ = *0.76*). A similar effect is observed in gamma band activity where IMC did not significantly change with perturbation (not presented) (*F(1.25, 19*) = *2.20, p* = *0.15*, η^2^ = *0.13*), while gamma band CMC (Fig 2D) did (*F(1.37, 19*) = *4.58, p* = *0.03*, η^2^ = *0.25*). Post-hoc analysis showed that gamma band CMC during F2 was significantly larger than those during F0 (*Z* = − *2.07, p = 0.04, r* = −*0.46*). The coherence between different pairs across trials is depicted in Supplementary Fig 2.

### 3.2 Sensorimotor beta bursts increased beta band corticomuscular coherence (CMC) and intermuscular coherence (IMC) across different muscle pairs, but only during static posture holding

**Fig. 3:**
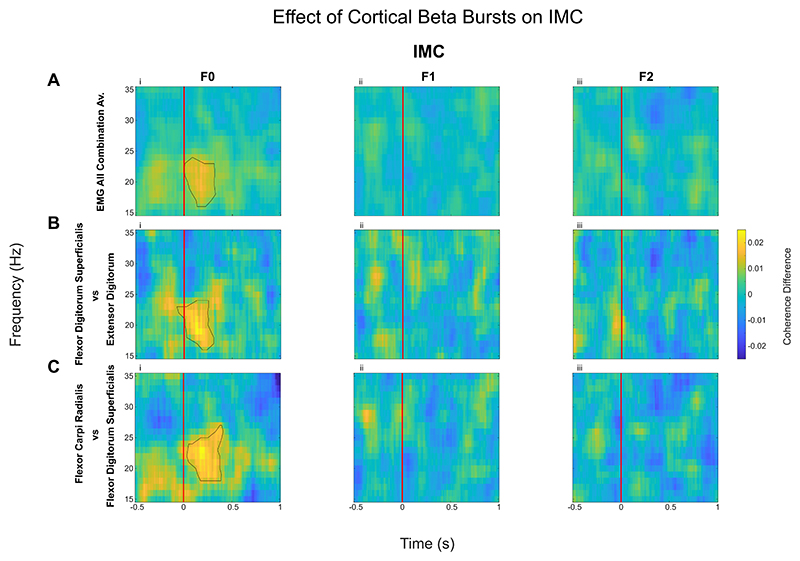
Intermuscular Coherence (IMC, quantified as PLV) increased with the onset of cortical beta bursts compared to no-burst, but only in natural posture holding (F0 condition). Coherence is calculated by finding the phase difference between the signals and then calculating vector strength over 250 ms periods (see Methods 2.5.4). Difference in IMC when aligned with the onset of cortical beta burst (time 0, shown as the red line) and ‘no-burst’ condition is presented here for the average across all recorded EMG pairs (A) and between flexor digitorum superficialis and extensor digitorum (Flx2 vs Ex2, B), as well as between flexor carpi radialis vs flexor digitorum superficialis (Flx1 vs Flx2, C), as an example. Contours show the statistically significant clusters found with permutation cluster analysis between burst-aligned and no-burst-aligned heatmaps (*p* < 0.05).

**Fig. 4:**
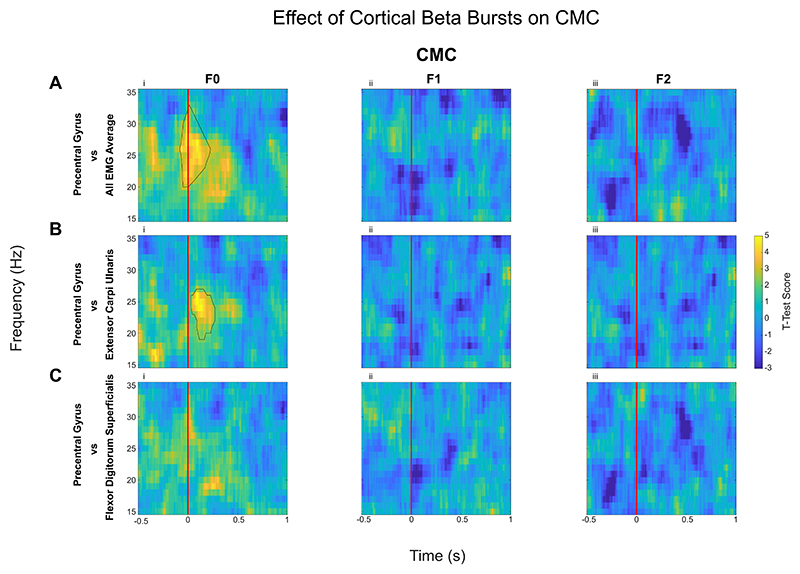
Corticomuscular Coherence (CMC, quantified as PLV) increased with the onset of cortical beta bursts compared to no-burst, but only in natural posture holding (F0 condition). Difference in CMC between contralateral precentral gyrus when aligned with the onset of cortical beta burst (time 0, shown as the red line) compared with ‘no-burst’ condition is shown here. The presented spectrograms demonstrate the average effect of the beta bursts on the CMC: the average of contralateral precentral gyrus with the average EMG (A), the precentral gyrus with extensor carpi ulnaris (B), and with flexor digitorum superficialis (C), as examples. Contours show the statistically significant clusters found with permutation cluster analysis between burst-aligned and no-burst-aligned heatmaps (*p* < 0.05).

**Fig. 5:**
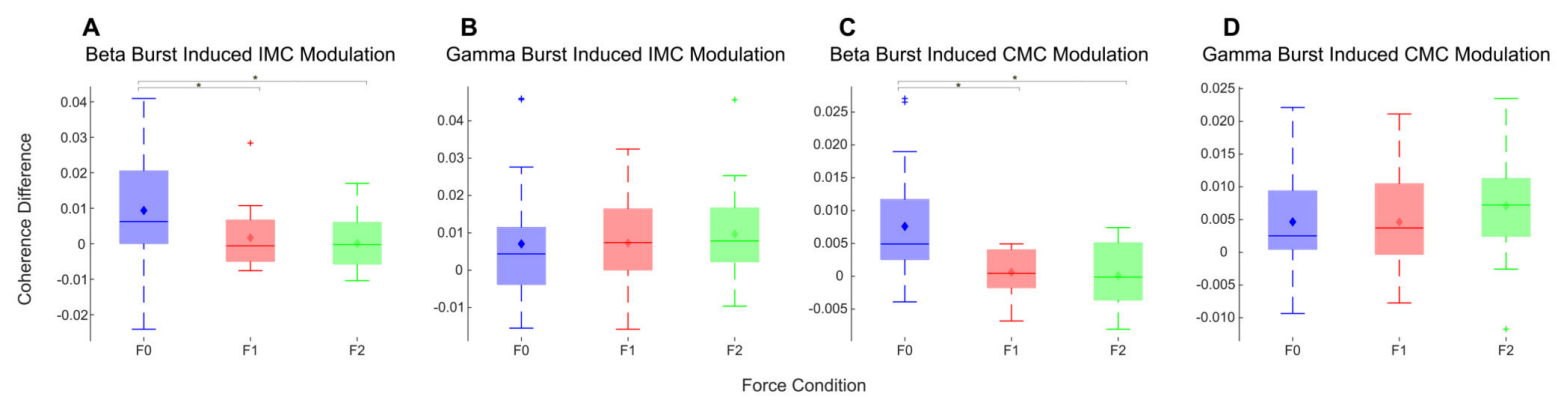
Perturbation forces in the steady hold phase modulate the effect of cortical beta bursts on the CMC and IMCs (quantified as PLV) compared with no-bursts. The cortical beta bursts increased the beta band IMC and CMC during natural posture holding (F0 condition), and the effect is larger than during dynamic perturbation resisting (F1 and F2 conditions). There is no such effect of cortical gamma bursts, and cortical gamma bursts didn’t lead to significant changes in CMC or IMC in any of the conditions. * indicates a significant difference in the post-hoc Wilcoxon signed rank test.

The effect of the cortical beta bursts on the muscle activity and synergy (intermuscular coherence) were evaluated by computing the IMC (Fig. 3) and CMC (Fig. 4) time locked to the onset of the beta bursts which was compared against no-burst observations, described in detail in Methods 2.5. This identified significant increase of IMC between different muscle groups in the beta frequency band during cortical beta bursts compared to no-burst condition. However, this was only the case for the F0 condition, when the participants were holding the handle in position (static posture holding) with no perturbation. Even though the average beta power is reduced during the Steady phase when participants had to resist unpredicted perturbations, beta bursts can still be detected. The results indicate that the cortical beta bursts resulted in a brief increase of phase locking between different muscle pairs during static posture holding, but not when the muscles need to be separately controlled to resist forces to different directions, as in F1 and F2 conditions, confirmed statistically with an ANOVA (*F(1.40, 19*) = *4.11, p* = *0.04*, η^2^ = *0.23*). Post hoc testing reveals cortical beta bursts led to larger increase in the beta band IMC in F0 compared with *F1* (*Z* = *2.07, p* = *0.04, r* = *0.46*) and F2 (*Z* = *2.43, p* = *0.04, r* = *0.54*). Similarly, CMC analysis revealed that in the F0 condition there was a significant increase in CMC in the beta band during cortical beta bursts compared with no burst condition (Fig. 4), while there was no effect on the CMC in F1 and F2. An ANOVA applied to the differences in beta band CMC (*F (1.37, 19*) = *9.56, p* = *0.002*, η*2* = *0.41*) induced by cortical beta bursts across the three force conditions (Fig. 5), reveals that there is a significant difference between the force conditions. Post hoc analysis revealed that, during cortical beta bursts, CMC is greater in F0 compared with F1 (*Z* = *2.99, p* =.*006, r = 0.67*) and F2 (*Z* = *2.91, p* =.*004, r* = *0.65*).

**Fig. 6:**
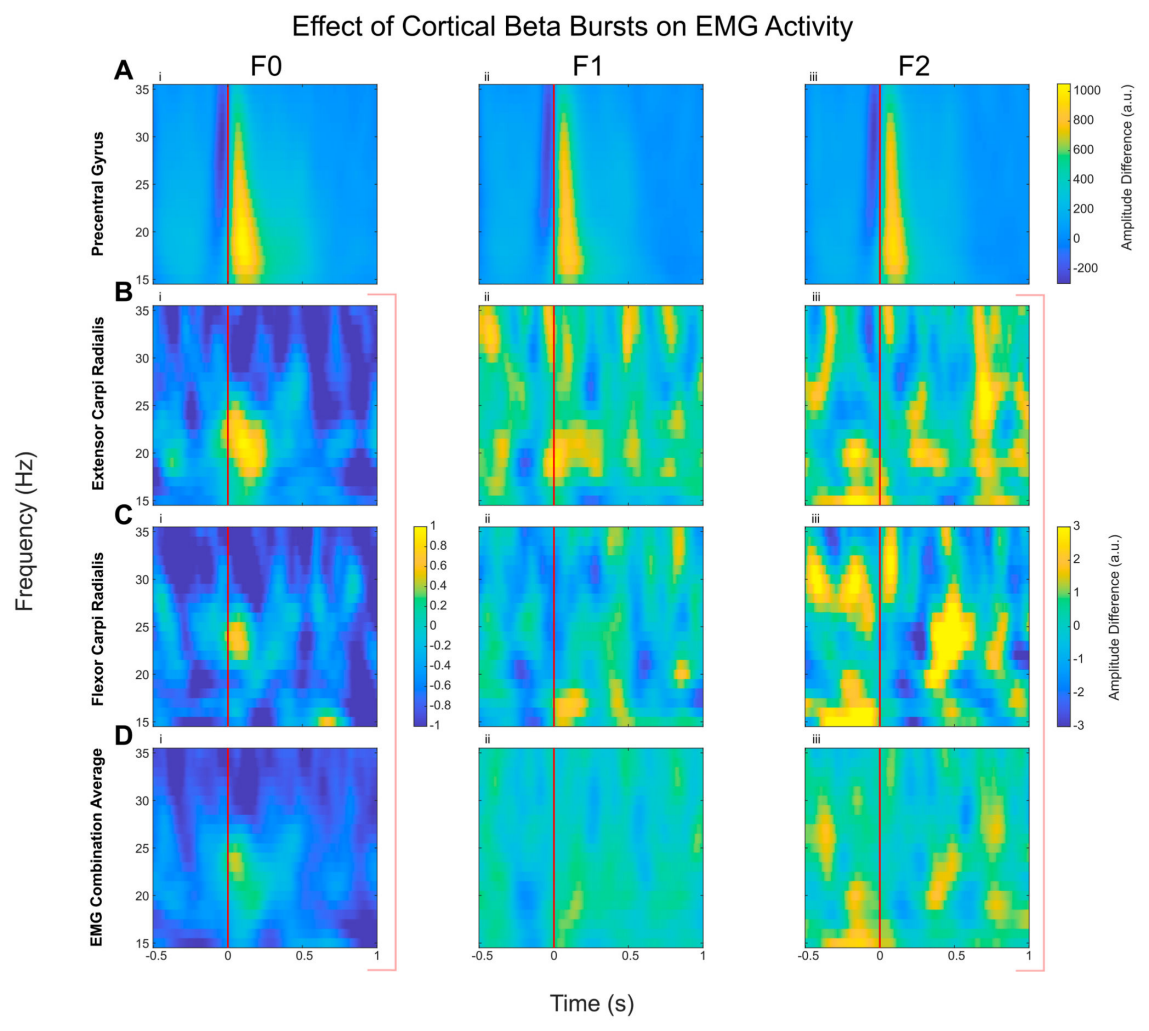
No significant increase in the EMG activities with the onset of cortical beta bursts compared to no-burst. The plots here show the difference in the power spectra of EMGs when aligned with the onset of cortical beta burst (time 0, shown as the red line) compared with ‘no-burst’ condition (for extensor carpi radialis (B), flexor carpi radialis (C), and average across all 4 recorded EMGs for each participant (D)). The vertical red line at 0 s denotes the onset of the beta burst (exceeding 75th percentile for a minimum duration of 100 ms) in the cortex. There seem to be increases in beta band activities in some individual muscles in the F0 condition (as shown in B), which echo results from the literature (Echeverria-Altuna et al., 2022). However, none of the results were found to be statistically significant, hence there are no contoured clusters.

Similar methods (found in Methods 2.5) were utilised to investigate how cortical beta bursts modulate EMG amplitude. This analysis demonstrated a trend of increased EMG activity in the beta frequency band in some individual muscles (Fig. 6 and Supplementary Fig 3), for example extensor carpi ulnaris (Fig. 6B (i)), especially in the F0 condition when there was no perturbation, however, this activity did not survive permutation based statistical testing.

### 3.3 The different effects of cortical beta bursts in CMC and IMC for different perturbation conditions are not due to potential difference in the burst amplitude or duration

Further investigation was conducted to determine whether the different effect of cortical beta burst on CMC and IMC during posture holding and active resisting was due to the different characteristics of beta bursts during these conditions.

As expected from the results shown in Figs. 1 and 3, beta bursts had larger duration, larger amplitude, and happened more often during F0 (static posture holding) compared to those during active resisting, despite using the same threshold to detect bursts for all conditions. A repeated measures ANOVA was conducted to test the effect of force condition on the amplitude, duration, and number of occurrences of cortical beta bursts, which all returned significant (*p < 0.05*). The amplitude of cortical beta bursts in F0 (*μ* = *1.79 a.u*.) was significantly larger than F1 (*μ* = *1.73 a.u*.) (*Z* = *3.21, p* =.*003, r* = *0.72*), and F2 (*μ* = *1.74 a.u*.) (*Z* = *2.58, p*=.*02, r* = *0.58*), with no differences between F1 and F2 (*Z* = −*1.16, p* =.*25, r* = −*0.26*). The duration of cortical beta bursts in F0 (*μ* = *231*.2 ms) was also significantly greater than F1 (*μ* = *201*.7 ms) (*Z* = *3.55, p<.001, r* = *0.79*), and F2 (*μ* = *204*.7 ms) (*Z* = *3.25, p* =.*002, r* = *0.73*), with no differences between F1 and F2 (*Z* = −*1.08, p* =.*28, r* = −*0.24*). The mean number of beta burst occurrences per participant in F0 (*μ* =*220.2* ± *26.1 bursts*) was significantly larger than F1 (*μ* =*161.6* ± *34.4 bursts*) (*Z* = *3.88, p <.001, r = 0.87*) and F2 (*μ* =*168.6* ± *49.1 bursts*) (*Z* = *3.30, p <.001, r 0.74*), with no differences between F1 and F2 (*Z* = −*1.29, p* =.*20, r* = −*0.29*).

To test whether the difference in the effect of beta bursts on IMCs between the different movement conditions was caused by difference in the beta burst duration or amplitude, the cortical bursts in F0 condition were ordered according to amplitude or duration for each participant and grouped into ‘low/high-amplitude beta bursts’ and ‘low/high-duration beta bursts’ by a median split (F1 and F2 were not median split). A repeated measures ANOVA was conducted to test for differences in the low amplitude F0 and normal amplitude F1 and F2 groups (which was repeated for duration) for both CMC and IMC with all returning a statistically significant result (*p <.05*). The ‘low-amplitude cortical beta bursts’ in the F0 condition had lower burst amplitude (*μ* = *1.44 a.u*.) and ‘low-duration cortical beta burst’ had lower duration (*μ* = *145*.7 ms) than those ‘normal cortical beta bursts’ during F1 (*Z* = *3.85, p <.001, r* = *0.86*) and F2 (*Z* = *3.85, p <.001, r* = *0.86*). When only the ‘low-amplitude beta bursts’ during the F0 conditions were considered, the difference in the IMC (between burst and no-burst) in F0 condition was still higher than the IMC difference during the normal bursts in F1 (*Z* = *2.13, p* =.*03, r* = *0.48*) and F2 (*Z* = *2.21, p* =.*03, r* = *0.49*). Furthermore, IMC difference induced by low-duration beta bursts during F0 was also higher than those normal beta bursts associated changes in F1 (*Z* = *2.21, p* =.*03, r* = *0.49*) and F2 (*Z* = *2.13, p* =.*03, r* = *0.48*). For CMC, the low-amplitude cortical bursts during F0 led to significantly greater increase in CMC compared to the normal bursts in F1 (*Z* = *3.06, p* =.*002, r* = *0.75*) and F2 (*Z* =*3.10, p* =.*002, r* = *0.69*). Similarly, the low-duration beta bursts during F0 led to higher increase in CMC than those normal duration bursts in F1 (*Z* = *2.69*, *p*=.*014, r* = *0.60*) and F2 (*Z* = *2.54, p* =.*01, r = 0.57*) conditions. Furthermore, to test whether the occurrence rate influenced the statistical results (the effect of the burst on IMC), observations were subsampled from the original dataset. Equal numbers (100) of bursts were randomly subsampled for each condition and each participant, to compare the effect of the bursts on IMC for different conditions. This procedure was then repeated 100 times to produce average results. This procedure did not change the significance of the statistics or the figures (Supplementary Fig 5).

Furthermore, plots of the manipulandum velocity, and the total error from the centre, aligned to the cortical beta burst onset are presented in Supplementary Fig 4, visually suggesting there is no obvious relationship between the burst and the manipulandum behaviour.

### 3.4 Cortical gamma bursting does not increase IMC

Gamma oscillations in the sensorimotor cortex tend to increase with voluntary movements, and increased gamma band CMC in this study was observed when participants needed to resist large perturbation forces. Therefore, whether gamma oscillation has a similar effect on increasing CMC and IMC, or whether this effect is specific to beta was also investigated. No

EMG amplitude or IMC increase was observed at the time of a cortical gamma burst. This was the same for all the three perturbation conditions, as there was no effect of perturbation forces on the cortical gamma burst induced changes in the IMC or CMC, which is confirmed by ANOVA *(F(2, 19*) = *0.20, p* = *0.82*, η*2* = *0.02 for IMC changes and F(1.55, 19*) = *0.66, p* = *0.53*, η*2* = *0.05 for CMC changes, respectively*).

## 4 Discussion

The presented experiment shows that beta bursts in the sensorimotor cortex are associated with increased phase locking value between the sensorimotor cortex and muscles, as well as between different muscles in the beta frequency band during posture holding without perturbation. However, this effect of cortical beta bursts was not observed when extra muscle activation was required to resist perturbation from unpredictable directions. This work builds on previous studies that demonstrate synchronisation between the motor cortex and motoneurons in the beta frequency band (Conway et al., 1995; Halliday et al., 1998; Salenius et al., 1996). However, most previous studies quantified average coherence over time during stable muscle contraction. Here, it is revealed that transient changes in the CMC and IMC are associated with transient increases in the cortical beta amplitude (beta bursts) even during stable motor output (static posture holding). Furthermore, this work indicates the role of bursting intervals is contingent on movement context (i.e. even when beta is high it has varying roles).

Recent studies, focusing on the temporal dynamics of neural signals, have revealed that beta oscillations may consist of transient bursting episodes that last a few cycles rather than sustained oscillatory activities (Ede et al., 2018; Feingold et al., 2015). The ‘burst’ interpretation has far-reaching implications about the nature of neural oscillations. Do the oscillations happen as isolated burst-events or are they sustained phenomena with dynamic amplitude variations, with high power bursting events defined as bursts on top of background tonic oscillations? For the case of beta oscillation in the sensorimotor cortical-basal ganglia network, single trial analysis of LFPs recorded from striatum and motor-premotor cortex in healthy monkeys showed that brief bursts of oscillation with the duration of 50–150 ms are responsible for virtually all beta-band activity, and that most of the modulations in trial-averaged beta power primarily reflect modulations of burst density (Feingold et al., 2015). This is consistent with results from healthy human participants showing that high-power beta events from somatosensory and frontal cortex typically lasted <150 ms and had a stereotypical non-sinusoidal waveform shape (Sherman et al., 2016). These observations support the hypothesis that physiological beta oscillations in the sensorimotor network happen as transient, isolated burst-events in healthy normal function.

Upper limb posture holding in the presence of dynamic unpredictable perturbations was associated with increased overall muscle activity to keep stability in healthy participants. This was accompanied with reduced beta oscillation in the sensorimotor cortex compared to static posture holding without perturbation. Even though beta bursts can still be detected during dynamic perturbation resisting, they don’t have a statistically significant impact on the CMC or IMC, compared to the low-beta period. The difference in the effect of cortical beta bursts across conditions doesn’t seem to be caused by the difference in the occurrence rate, average amplitude or duration of the cortical beta bursts. The findings provide experimental evidence for the hypothesis that senso-rimotor beta bursts can help coordinate muscles and promote muscle synergy, therefore beta bursts may play an important role in posture holding. Meanwhile, the results also suggest that the relationship between cortical beta bursts and muscle activities change with the behavioural context.

### 4.1 The role of cortical beta bursts in healthy motor control

IMC reflects functional coordination between different muscles during specific tasks or movements (Laine and Valero-Cuevas, 2017). IMC plays an influential role in posture holding, where increased IMC reflects the maintenance of stability and balance. The presented experiment demonstrates that during upper limb posture holding without perturbation, cortical beta bursts were associated with increased CMC and IMC in the same frequency band, even though no statistically significant increases in the beta band amplitude in the EMG measurement from individual muscles were observed. There was also no statistically significant effect of cortical beta bursts in CMC and IMC in the conditions when participants had to resist dynamic perturbations to keep stable.

The lack of effect of cortical beta bursts on EMG amplitude is different from findings in (Echeverria-Altuna et al., 2022) where beta band increase was also observed in the muscle activities with the occurrence of beta bursts in the cortex when the participants were asked to generate stable gripping force. This difference in observations could possibly be due to smaller overall muscle activity in the F0 condition in the presented task, which is more similar to static posture holding where stable force is not demanded. This could be tested with an experiment involving a task with three conditions, where the participant is required to grip a device with the forearm (isometric contraction) and apply no force, small force, and a large force while maintaining a static posture hold. This setup would help determine whether the lack of cortical beta burst effect on EMG amplitude is due to the amount of force generated by the muscle.

The findings presented in this study provide compelling evidence of the role of beta bursts in increasing IMC in healthy motor control, particularly during natural posture holding with no perturbation. These findings support suggestions that beta bursts play a crucial role in synchronising the phases of different elements of the muscle network responsible for posture stabilisation, aligning with theories that beta bursts represent brief periods of interregional communication (Little et al., 2019; Lundqvist et al., 2018). However, this phenomenon was not observed when participants had to resist fast and unpredictable perturbations, which may be achieved by isotonic movements of individual muscles which changes over time depending on the direction of the perturbation. In this condition, the average beta power reduced in the sensorimotor cortex, and the overall CMC in the beta band reduced compared to the natural static posture holding condition. Even though transient increase of beta can still be observed, they don’t have any effect on the muscle activity or IMC. These results suggest that the role of cortical beta bursts in synchronising functionally connected muscles may be specific to static posture holding or isometric contractions. During dynamic movements, the corticospinal oscillation mode of the sensorimotor system shifts towards higher (principally gamma) frequencies, consistent with previous studies (Gwin and Ferris, 2012; Omlor et al., 2007). It has been suggested that tasks requiring increased integration of visual and somatosensory information may shift the frequency of the corticomuscular coherence to the gamma-range (Omlor et al., 2007). Muscle dynamics, including the amount and type of proprioception, may play a role in the beta-to-gamma shift.

### 4.2 Implications for Parkinson’s disease

Patients with Parkinson’s disease are not only impaired in movement initialisation, they are also impaired in relaxing a contraction (Jordan et al., 1992; Kunesch et al., 1995; Neely et al., 2013; Robichaud et al., 2005; Stelmach and Worringham, 1988; Wing, 1988), halting, correcting or decelerating a movement (Angel et al., 1970; Manza et al., 2017; Poon et al., 2011). Therefore, it has recently been suggested that motor impairment in Parkinson’s disease includes a general impairment in transitioning between stable and dynamic movement states (Herz and Brown, 2023). A bias towards the stable state is consistent with the observed rigidity in Parkinson’s disease, i.e., muscle co-contractions that reinforce a postural state (Mazzoni et al., 2012). Similarly, in optimal feedback control theory, rigidity could be interpreted as a motor control dysfunction in which the limbs are programmed to be excessively stable (Mazzoni et al., 2012). The presented work indicates that during natural posture holding, cortical beta oscillations may help coordinate muscles and beta bursts are associated with transient increase of phase synchrony across muscle groups. This may be exaggerated in Parkinson’s disease leading to rigidity which could help explain why restoring physiological beta activity modulation in Parkinson’s disease improves patients’ ability to flexibly adapt their behaviour (Brown, 2006; Doyle et al., 2005).

There are fewer studies focussing on how muscle coordination was impaired or whether intermuscular coherence was abnormal in PD. In one study, increased IMC during an isometric wrist extension and finger abduction task was linked to improvements in bradykinesia in some cases, driven by successful coordination of muscles through STN DBS (Brown et al., 2001). However, in other cases, increased IMC has been observed in PD, potentially associated with rigidity (Flood et al., 2019). These seemingly opposing views can be reconciled by considering different contexts and motor control mechanisms. In the presented work, there was reduced beta in the sensorimotor cortex during posture holding with perturbation compared to without perturbation in the healthy subjects. Therefore, the increased muscle-coaction induced by perturbation in healthy adults in this study can be very different from the rigidity observed in Parkinson’s disease.

It is important to the study of pathology in movement disorders to probe the effect of cortical beta bursts on the stability and balance of integrated muscle networks in Parkinson’s patients, particularly to observe the differences with healthy beta bursts and better define the disease.

### 4.3 Limitations and future work

The study is limited to only 20 participants and how well the observed effects translate to the wider population remains unclear. A further limitation of the study is that the EMG activity in condition F0 is low and has large cross participant variation which may explain the large variance in the IMC values in this condition as shown in Fig. 5A. The amount of force applied by the participant in F0 is variable, whereas in F1 and F2 the paradigm forces them to stabilise in a similar way.

It would also be interesting to observe whether EMG power and coherence patterns change systematically in response to different perturbation directions, which future work should aim to address. Furthermore, it will be important to establish the differences between Parkinsonian and healthy beta bursts in a similar context. Do pathological beta bursts also generate coherence between different muscle groups? Or does pathological activity impair the healthy mechanism? This would help to further understand the role of beta bursts in movement disorders, and potentially enable better treatments.

## Supplementary Material

Supplementary material associated with this article can be found, in the online version, at doi:10.1016/j.neuroimage.2024.120774.

Supplementary Material

## Data Availability

The datasets produced and analysed in this study are available on https://data.mrc.ox.ac.uk/ for free access.
