# Supplementary material for: Cortical beta oscillations help synchronise muscles during static posture holding in healthy motor control
Source: Neuroimage. 2024 Sep;298:120774. doi: 10.1016/j.neuroimage.2024.120774 (PMC7617462; doi:10.1016/j.neuroimage.2024.120774)
Supplement: Supplementary file 1 [file mmc1.docx]

Cortical beta oscillations help synchronise muscles during static posture holding in healthy motor control – Supplementary Figures


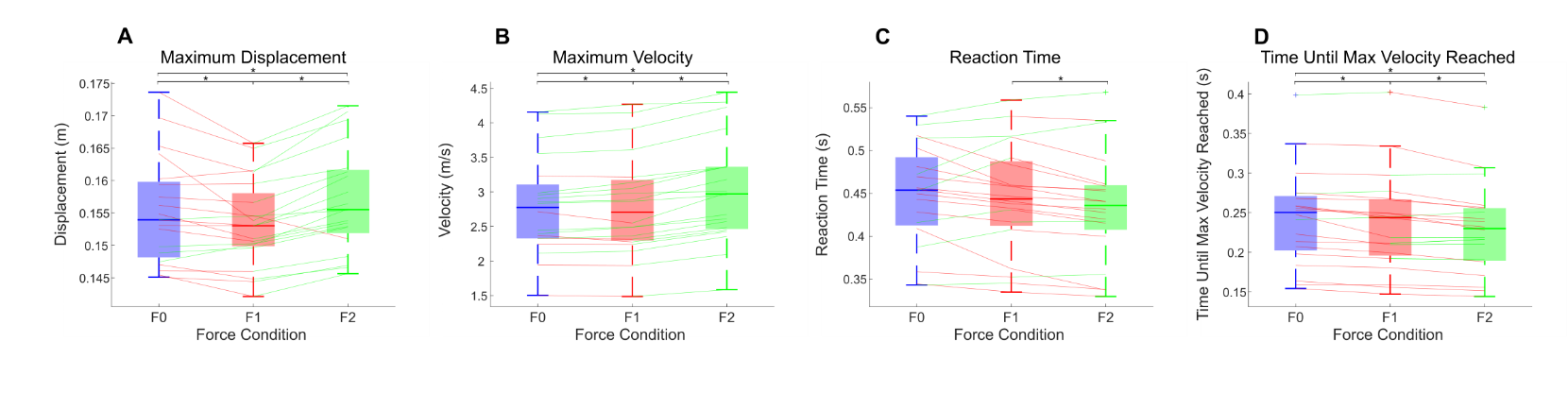


Supplementary Figure 1: Box plots of behavioural metrics in different force conditions from the reaching phase only. Lines on the plot indicate different participants, where red indicates a decrease and green indicates an increase in value. * indicates a significant difference in the post-hoc Wilcoxon signed rank test.


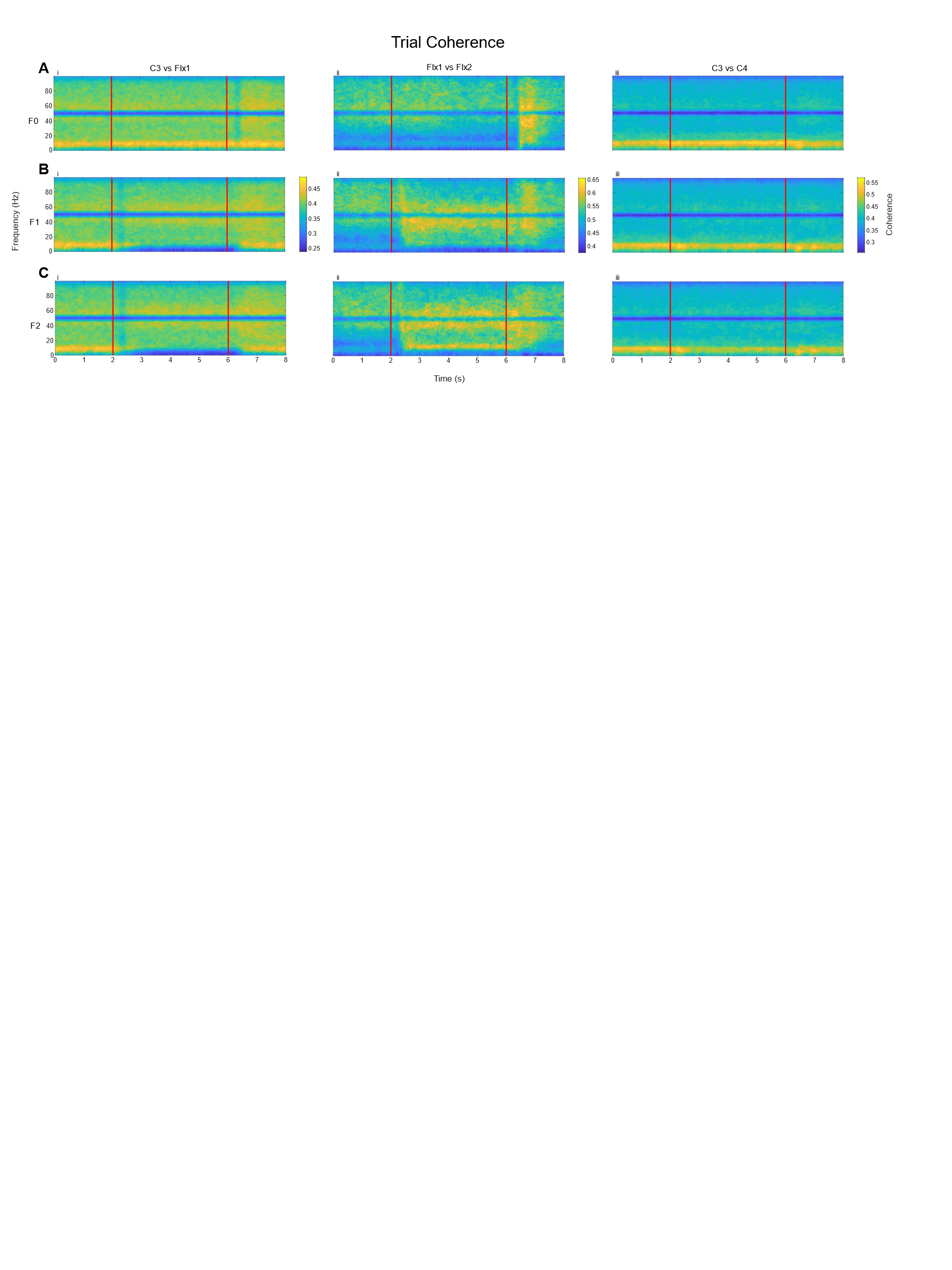


Supplementary Figure 2: Average coherence between C3 vs Flx1, Flx1 vs Flx2, and C3 vs C4 during the trial. Coherence is calculated as described in Methods 2.5.4


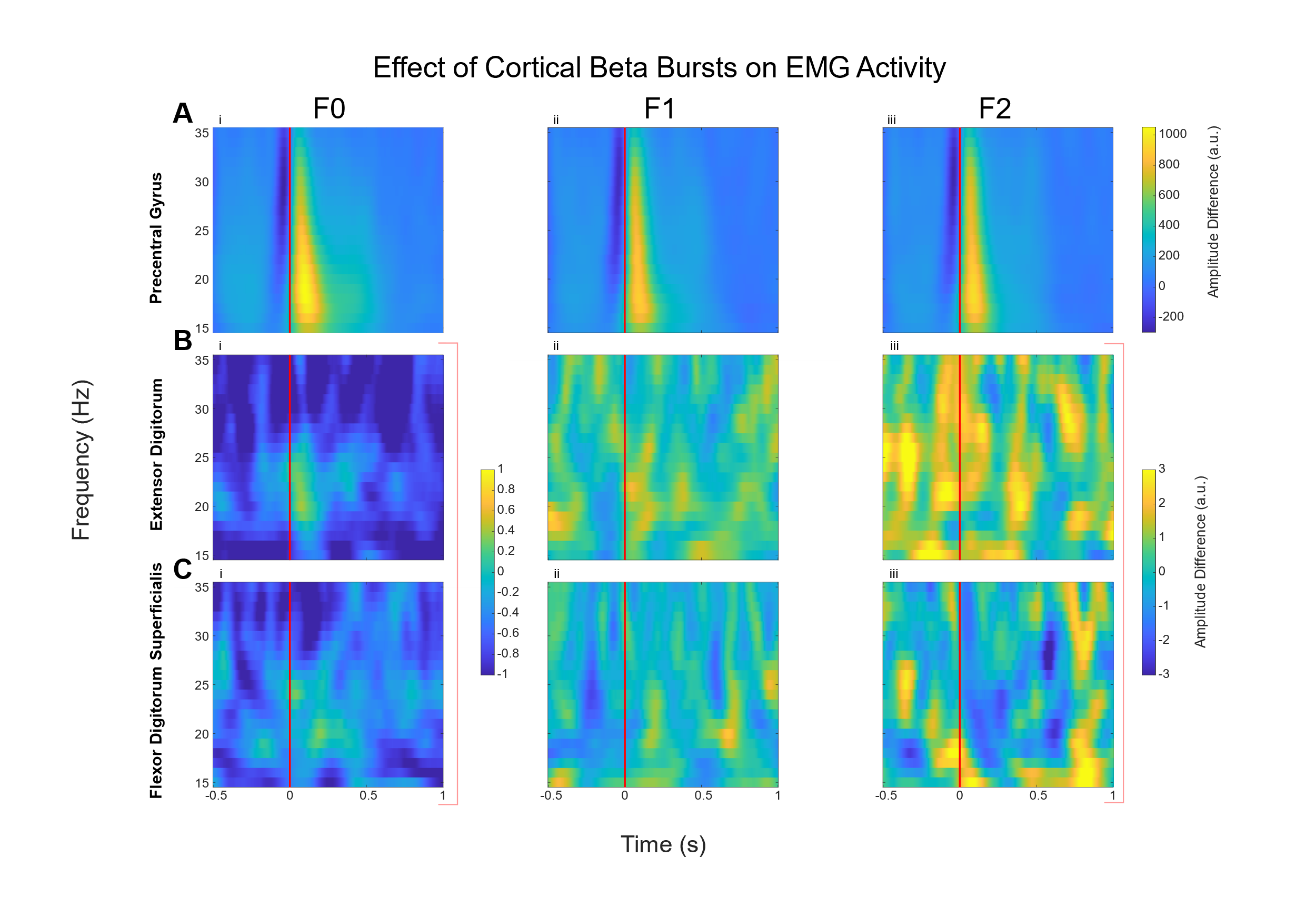


Supplementary Figure 3: Spectrogram demonstrating beta amplitude in precentral gyrus and EMG aligned to the onset of the cortical beta burst. Beta amplitude in extensor digitorum and flexor digitorum superficialis are demonstrated. The vertical red line at 0s denotes the onset of the beta burst (exceeding 75^th^ percentile for a minimum duration of 100ms) in the cortex. None of the results were found to be statistically significant, hence there are no contoured clusters.


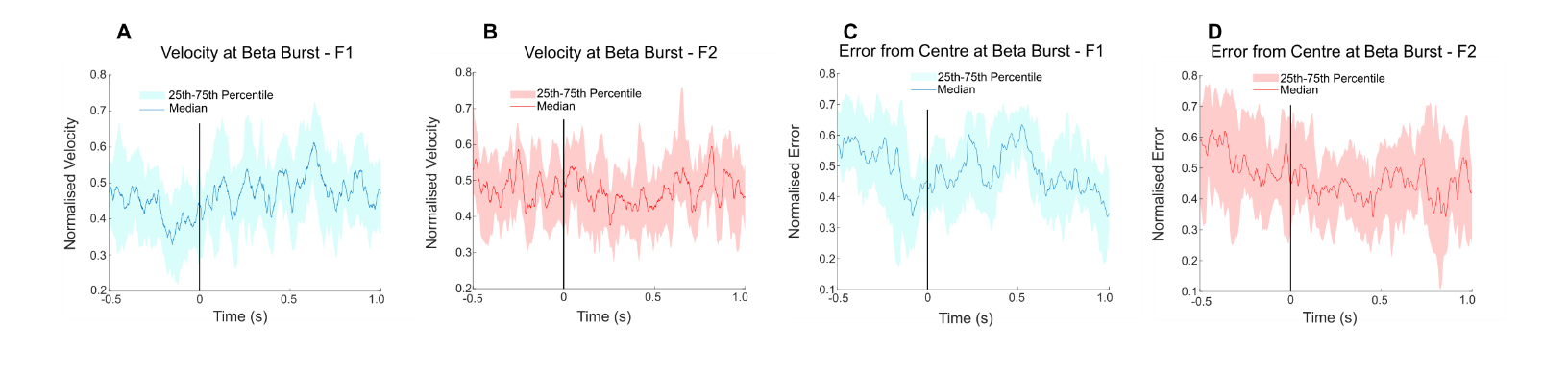


Supplementary Figure 4: Manipulandum statistics around the bursting period.


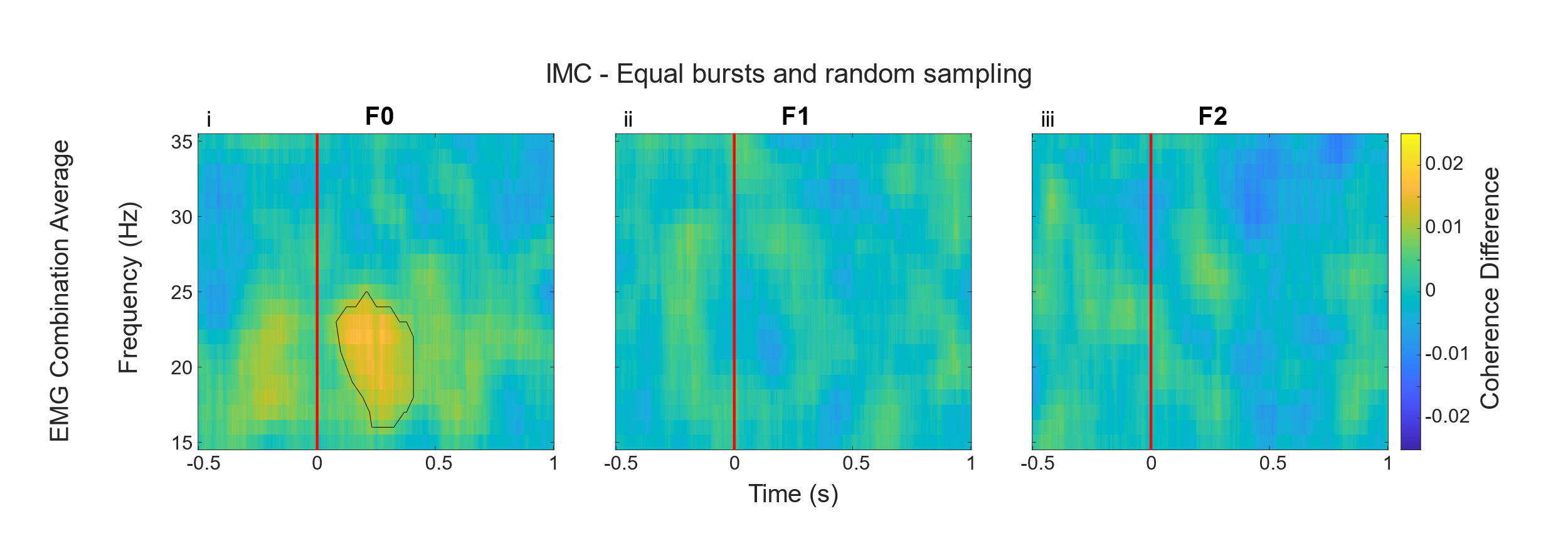


Supplementary Figure 5: IMC averages with equal numbers of bursts subsampled from each force condition and each participant.


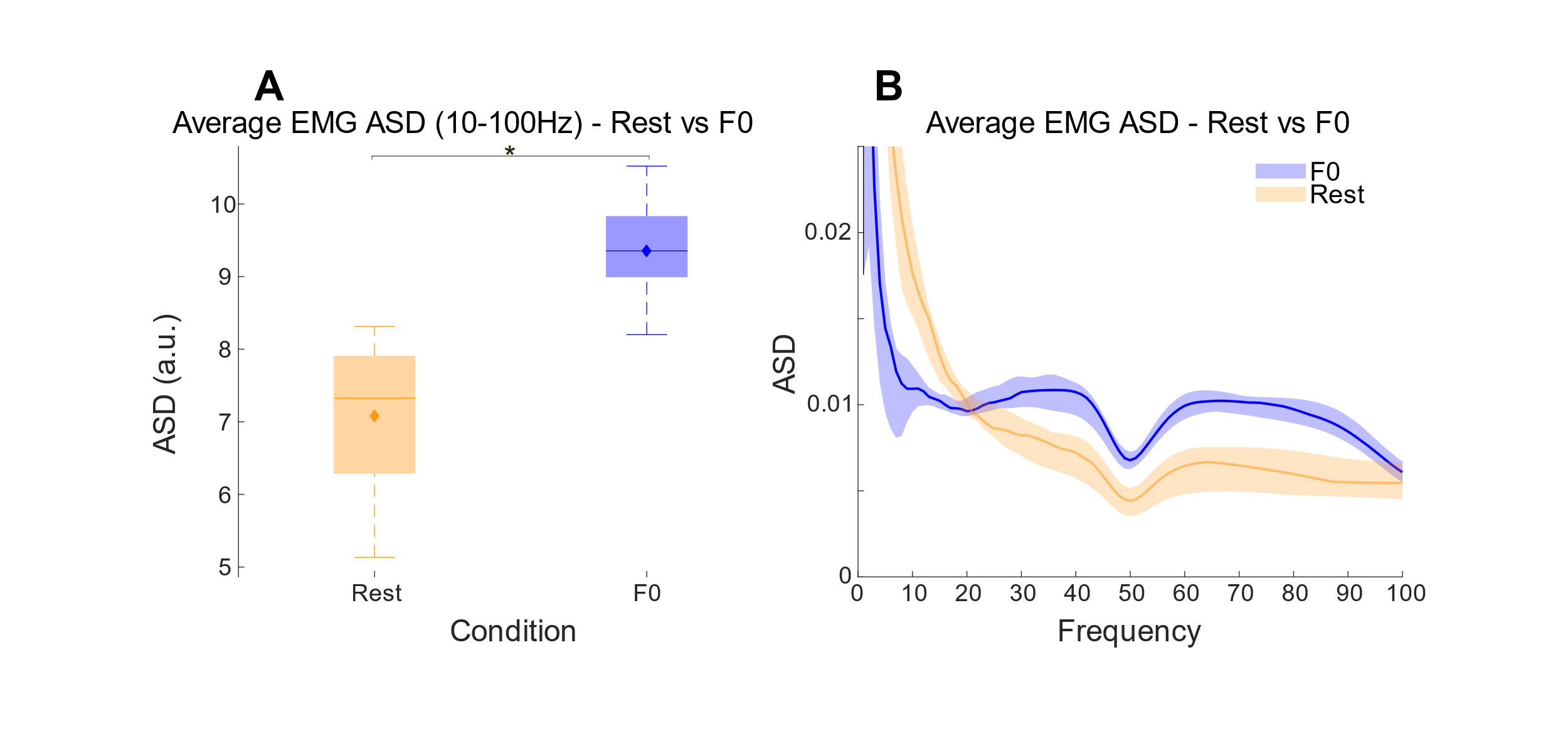


Supplementary Figure 6: (A) Average EMG Amplitude Spectral Density (ASD) boxplot and (B) periodogram, demonstrating the differences between F0 and rest condition. Rest condition refers to complete rest, recorded prior to the paradigm (not the rest period in the paradigm).
